# Association Between US Drug Price and Measures of Efficacy for Oncology Drugs Approved by the US Food and Drug Administration From 2015 to 2020

**DOI:** 10.1001/jamainternmed.2022.4924

**Published:** 2022-10-31

**Authors:** Miloš D. Miljković, Jordan E. Tuia, Timothée Olivier, Alyson Haslam, Vinay Prasad

**Affiliations:** 1 Cartesian Therapeutics, Gaithersburg, Maryland; 2 Department of Epidemiology and Biostatistics, University of California, San Francisco; 3 Department of Oncology, Geneva University Hospital, Geneva, Switzerland; 4 Department of Medicine, University of California, San Francisco

## Abstract

This cross-sectional study estimates all US Food and Drug Administration anticancer approvals in recent years and evaluates if an association exists between their cost and efficacy.

The US has worse cancer-related outcomes than other high-income countries while bearing the highest cost of cancer care in the world.^1,2^ A reason for increasing cost may be improved efficacy of expensive novel agents. One study found an association between measures of benefit and price, although the study considered gains in progression-free survival (PFS) to be the same as gains in overall survival (OS),^3^ an assumption that may be debated, given the uncertainty regarding health benefits associated with PFS.^4^ Another study of all cancer drug approvals from 2009 to 2013 found no association between measures of efficacy and pricing of cancer drugs.^5^

The past 5 years have seen a marked increase in the number of cancer therapies on the market and a greater interest in value-based pricing. It is unclear whether the value proposition has shifted: have cancer drugs started to rely on improved benefit and decreased use of surrogate end points to justify skyrocketing costs? We described these trends by estimating all US Food and Drug Administration (FDA) anticancer approvals in recent years and evaluating if an association exists between their cost and efficacy.

## Methods

In this retrospective, cross-sectional analysis of all cancer drugs approved by the FDA from January 1, 2015, to December 31, 2020, we assembled metrics of their activity or efficacy and their cost per annum or course of treatment as previously described.^5,6^ All prices were rounded to the nearest $1000. For categorical variables, the Kruskal-Wallis test was used to determine significance differences between median annual cost of subgroups, with *P* < .05 considered statistically significant. Linear models for the continuous variables of annual cost and percentage of improvement were stratified by end points. Analysis was conducted in R, version 4.1.2 (R Foundation). The search was performed in December 2021. Because we used publicly available data, and this was not human participants research, we did not submit this study to an institutional review board.

## Results

We analyzed 224 FDA oncological drug approvals across 119 individual drugs. Across all tumor types, the median annual cost for a course of an oncology drug was $196 000 (IQR, $170 000-$277 000; Table).

**Table.** Characteristics of US Food and Drug Administration Oncology Drug Approvals From 2015 to 2020

Characteristic	Overall, No. (%)
No.	224
Annual cost, median (IQR), $	196 000 (170 000-277 000)
Mechanism	
Small molecule therapy	102 (45.5)
Biologics	99 (44.2)
Cytotoxic therapy	11 (4.9)
Hormonal therapy	7 (3.1)
Gene and oncolytic virus therapy	5 (2.2)
Tumor type	
Other	106 (47.3)
Non–small-cell lung cancer	32 (14.3)
Breast	21 (9.4)
Myeloma	14 (6.2)
Acute myeloid leukemia	12 (5.4)
Melanoma	11 (4.9)
Urothelial	10 (4.5)
Ovarian	9 (4.0)
Prostate	9 (4.0)
Primary end point	
Overall response rate	90 (40.2)
Progression-free survival	71 (31.7)
Overall survival	46 (20.5)
Other	12 (5.4)
Disease-free survival	5 (2.2)
Target	
Other	129 (57.6)
Programmed cell death protein 1	45 (20.1)
Programmed cell death ligand 1	16 (7.1)
Poly-ADP ribose polymerase	13 (5.8)
Pan-kinase	11 (4.9)
Cell cycle	10 (4.5)
Novelty of drug	
Subsequent approvals of the same drug	140 (62.5)
First approvals of a next-in-class drug	55 (24.6)
Approved based on a new mechanism of action	29 (12.9)

We detected a significantly lower median annual cost of drugs whose approval was based on OS ($185 000; IQR, $159 000-$206 000; n = 46) compared with those with PFS-based ($203 000; IQR, $183 000-$248 000; n = 71; *P* = .02) or overall response rate (ORR)–based approvals ($239 000, IQR, $185 000-$341 000, n = 90, *P* < .01). Price had a weak correlation within the most common approval end points (*R*^2^ = 0.14 for OS, *R*^2^ = 0.16 for PFS, and *R*^2^ = 0.02 for ORR; Figure), ie, variability in efficacy only captured less than 15% of the variability in price. There was no significant difference between the median price of drugs approved after randomized clinical trials ($191 000) and those with no randomized clinical trial data ($206 000; *P* = .06).

**Figure.**
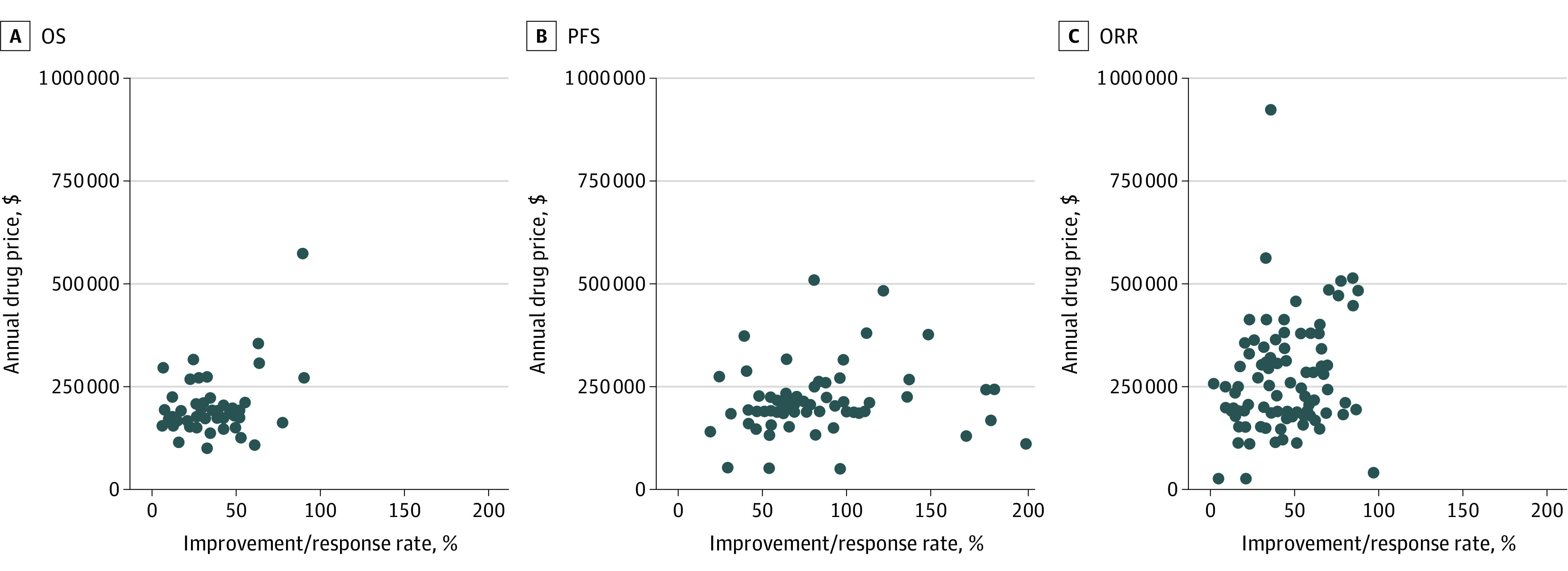
Median Annual Cost by End Point for US Food and Drug Administration Approved Drugs From 2015 to 2020 Outliers (n = 7) were excluded from the figure for clarity, and each dot represents 1 approved drug. ORR indicates overall response rate; OS, overall survival; PFS, progression-free survival.

## Discussion

In value-based pricing, drugs that are associated with greater improvements to the same end point or more improved objective end points (ie, PFS rather than ORR, OS rather than PFS) would be expected to cost more. The results of this analysis suggest the opposite: oncology drugs approved based on OS improvement had the lowest median annual cost of the 3 end points. This finding was similar to prior work that found a significantly higher median price of drugs with ORR-based approvals.^5^ Furthermore, we did not detect a meaningful association between cancer drug prices and the magnitude of benefit for any of the end points. Among the drugs approved on the basis of response rate, there was only a weak correlation between cost and the magnitude of the response rate gain; the same was true in the categories of drugs approved on the basis of PFS and OS gains. This suggests that cancer drugs are priced based predominantly on what the market will bear. Correcting this trend is vital for the solvency of health care and pharmaceutical development. Limitations of the study include restriction of analysis to a 6-year period, and restriction of analyzed factors affecting drug price to end points only.
